# Betriebliche Absichten für gesundheitsförderliche Telearbeit nach dem COVID-19-Lockdown 2020

**DOI:** 10.1007/s11553-022-00956-y

**Published:** 2022-06-16

**Authors:** Gert Lang, Kathrin Hofer-Fischanger

**Affiliations:** 1 Gesundheit Österreich GmbH, Fonds Gesundes Österreich, Wien, Österreich; 2 Institut Gesundheits- und Tourismusmanagement, FH JOANNEUM – University of Applied Sciences, Kaiser-Franz-Josef-Straße 24, 8344 Bad Gleichenberg, Österreich

**Keywords:** Homeoffice, Kapazitätsaufbau, Gesundheitsförderung, Unternehmenskultur, Multivariate Analyse, Home office, Capacity building, Health promotion, Organizational culture, Multivariate analysis

## Abstract

**Hintergrund:**

Unternehmen mussten wegen der Pandemie im Frühjahr 2020 schnelle Lösungen für die Weiterarbeit – von zu Hause aus – finden. Die Arbeitsbedingungen zu Hause (Telearbeit) entsprechen nicht immer den Grundprinzipien und Qualitätskriterien der betrieblichen Gesundheitsförderung (BGF).

**Fragestellung:**

Wie stark ist der Ansatz gesundheitsförderlicher Telearbeit (Arbeit im Homeoffice) in Betrieben verankert und was beeinflusst die strategische Etablierung und Nutzung unterstützender Materialien?

**Material und Methoden:**

Theoretisch abgeleitete Hypothesen wurden operationalisiert und 1858 österreichische Betriebe zur Teilnahme an einer Online-Befragung eingeladen. Die Stichprobe (*n* = 192) repräsentiert einen breiten Mix aus Unternehmensgrößen, Sektoren und Regionen.

**Ergebnisse:**

Betriebe variieren stark in der Absicht zukünftig gesundheitsförderliche Telearbeit umzusetzen. Ein Teil der Variation kann durch multivariate Pfadmodelle aufgeklärt werden, wobei die Verhaltenskontrolle und die sozialen Normen eine zentrale Rolle einnehmen. Erstere wird vom Grad der Vorbereitung auf Telearbeit und ihre Umsetzung im Unternehmen bestimmt. Insbesondere zeigt sich, dass die Telearbeitskultur und die ‑bereitschaft für die Stärke von sozialen Normen gegenüber der Umsetzung von Telearbeit verantwortlich sind.

**Schlussfolgerung:**

Telearbeit wurde bisher zu wenig im Sinne einer ganzheitlichen BGF betrachtet. Eine derartige Umsetzung hängt stark von Unternehmensstrukturen und -prozessen, der Kultur und den Handlungsspielräumen der Entscheidungsträger ab. Betrieben wird empfohlen, Kompetenzen aufzubauen und sich am Konzept der Kapazitätsbildung zu orientieren.

## Abstract (en)

**Background:**

Companies had to find quick solutions for continuing to work due to the pandemic in spring 2020. However, working conditions at home (teleworking) do not always comply with the basic principles and quality criteria of workplace health promotion (WHP).

**Objectives:**

How strongly is the approach of health-promoting telework (working at home) established in companies and what influences the strategic anchoring and the use of supporting materials?

**Materials and methods:**

Theoretically derived hypotheses were operationalised and 1858 Austrian companies were invited to participate in an online survey. The sample (*n* = 192) represents a broad mix of company sizes, sectors and regions.

**Results:**

Workplaces vary widely in their intention to implement health-promoting telework in the future. A part can be explained by multivariate path models, with behavioural control and social norms playing a central role. The former is determined by the degree of preparation for telework and its implementation in the company. In particular, teleworking culture, in addition to teleworking readiness, is shown to be responsible for the strength of social norms towards its implementation.

**Conclusions:**

Teleworking has so far received too little attention in the sense of holistic WHP. Such an implementation strongly depends on the health-promoting corporate structures and processes, the culture, and the decision-makers’ scope for action. Companies are recommended to follow the concept of capacity building in order to build up competencies and knowledge and to enable appropriate measures.

Der Ausbruch der COVID-19-Pandemie hat zu einer starken Zunahme von Telearbeit^1^ in vielen Betrieben geführt. Gesundheitsthemen von Beschäftigten im Homeoffice sind dabei stärker in den Fokus gerückt. Es ist jedoch wenig bekannt, unter welchen Bedingungen sich Betriebe für gesundheitsförderliche Gestaltung der Telearbeitsform einsetzen. Ein wichtiger Beitrag in der Gesundheitsförderung liegt beim Kapazitätsaufbau. Neben der strategischen Verankerung ist die Vermittlung von Know-how zur Gestaltung von Telearbeit und zum Aufbau oder zur Verbesserung von Strukturen und Arbeitsverhältnissen unumgänglich.

## Hintergrund und Fragestellung

### Zunahme von Telearbeit (TA)

Um die Ausbreitung des Virus einzudämmen, die Beschäftigung der erwerbstätigen Bevölkerung aufrechtzuerhalten und die negativen wirtschaftlichen Folgen der Pandemie zu begrenzen, stellten viele Betriebe und Arbeitgeber:innen im Frühjahr 2020 auf TA um. Während dieser Zeit erfuhr TA einen neuen Höhepunkt. Die International Labour Organisation (ILO) bezeichnete die rasante Entwicklung als einen „Teleworking-Tsunami“ [14]. Eine europäische Umfrage belegt, dass 48 % der befragten Beschäftigten während der COVID-19-Pandemie zumindest zeitweise, 34 % ausschließlich zu Hause gearbeitet hat [9]. Dabei liegen starke Unterschiede zwischen Ländern (z. B. Ökonomien), Wirtschaftsbereichen (wissensintensive Sektoren), Betriebsgrößen (Großbetrieben), Arbeitsorganisationen (autonome, flexible Arbeitszeitregelung) und Qualifizierung der Erwerbstätigen (digitale Kompetenzen) vor.

### Arbeitsbedingungen und Gesundheit bei Telearbeit

Die TA wird bisher unterschiedlich definiert, ein Definitionsversuch ist z. B.: „Teleworking occurs when employees perform all or a substantial part of their work physically separated from the location of their employer, using IT for operation and communication“ [4]. Die digitalen Informations- und Kommunikationstechnologien (IKT) in der zunehmend flexibilisierten (Arbeits‑)Welt machen eine Verbreitung von TA erst möglich. Die Bedingungen für das Arbeiten im Homeoffice sind jedoch der Gesundheit nicht immer zuträglich. Insbesondere berichten Studien über Auswirkungen der Arbeit im Homeoffice auf die körperliche, mentale und soziale Gesundheit [17, 19, 24]. Die vorliegende Evidenz zeigt, dass TA wesentliche gesundheitliche Vorteile für Mitarbeitende und wirtschaftliche Vorteile für Unternehmen bringt. Die negativen Auswirkungen können aber langfristig gesundheitliche Probleme bei Mitarbeitenden und Führungskräften begünstigen.

### Telearbeit und BGF

Die betriebliche Gesundheitsförderung (BGF) stellt im Rahmen des betrieblichen Gesundheitsmanagements (BGM) ein qualitätsvolles Konzept bzw. eine Strategie zur Unternehmens- bzw. Organisationsentwicklung dar. BGF verfolgt nach Luxemburger Deklaration^2^ das Ziel, die Gesundheit am Arbeitsplatz zu stärken, das Wohlbefinden der Beschäftigten zu verbessern und Krankheiten vorzubeugen [5]. Systematische Übersichtsarbeiten belegen ihre Wirksamkeit, v. a. wenn umfassende (multimodale, ganzheitliche) Programme auf Verhältnis- und Verhaltensebene umgesetzt werden [10, 23], auch bei niedrigeren sozioökonomischen Positionen [25].

Die BGF kann daher eine entscheidende Rolle für Unternehmen spielen, mit den neuen Herausforderungen in der Arbeitswelt umzugehen. Das trifft insbesondere auf die Phase des tiefgreifenden Wandels in der Arbeitswelt zu (z. B. Digitalisierung, Flexibilisierung), wo sich zentrale Rahmenbedingungen für Gesundheit und Arbeit verändern. Trotz alledem ist der gesundheitsförderlichen TA bisher wenig Aufmerksamkeit geschenkt worden [11].

Für die BGF stellt sich angesichts der stärkeren Verbreitung von TA die Frage nach den Absichten und Voraussetzungen für die Umsetzung einer gesundheitsförderlichen TA. Daher geht der Beitrag diesen Fragestellungen nach:

- Wie stark ist die Absicht von Betrieben, gesundheitsförderliche TA strategisch zu verankern und dabei unterstützende Materialien (z. B. einen Praxisleitfaden) zu verwenden?
- Welche Faktoren begünstigen oder hemmen diese Intentionen?

Auf Basis theoretischer Überlegungen wurde eine empirische Untersuchung durchgeführt. Der Beitrag diskutiert die Ergebnisse und identifiziert relevante organisationale Strukturen und Prozesse zum Einsatz und Ausbau von TA im Unternehmen.

## Studiendesign und Untersuchungsmethoden

### Theoretischer Hintergrund

Die hier intendierte Untersuchung der unternehmerischen Disposition zur gesundheitsförderlichen TA basiert auf den von Baruch [4] vorgeschlagenen organisationalen und arbeitsplatzbezogenen Faktoren von TA und berücksichtigt die damit im Zusammenhang stehenden Prozesse und Rahmenbedingungen (z. B. Machbarkeit, Vorbereitung, Vorerfahrung, Unternehmenskultur). Für die Erklärung TA umzusetzen wird auf die Handlungstheorie des geplanten Verhaltens („theory of planned behavior“ [TPB]) zurückgegriffen [1, 2]. Sie geht davon aus, dass Individuen (u. a. Beschäftigte, Führungskräfte) grundsätzlich durch ihre Handlungsmotivation und -absichten in ihrem Verhalten geleitet werden, überwiegend beeinflusst durch verschiedene Faktoren auf personaler, sozialer und organisationaler Ebene. Die Verhaltensintention ist Ergebnis (a) eines affektiven Bewertungsprozesses (Einstellung) bzw. der Überzeugung, dass das Verhalten zu bestimmten Ergebnissen führt, sowie der Bewertung dieser Ergebnisse (z. B. Nutzenerwartung), (b) einer subjektiven Überzeugung davon, was andere Personen erwarten und befürworten (soziale Norm), wie auch (c) die vom Individuum subjektiv wahrgenommene Schwierigkeit (Selbstwirksamkeit), ein Verhalten auszuführen (Verhaltenskontrolle). Der Ansatz hat Überlegungen zur Techniknutzung bzw. -akzeptanz [8], auch hinsichtlich der Umsetzung von TA, inspiriert [20].

Infolgedessen lautet die theoretische Grundannahme, dass die Umsetzung von gesundheitsförderlicher TA umso wahrscheinlicher ist, je stärker die Intention ihrer Realisierung ist. Aus der Perspektive der Organisationsentwicklung wird zunächst angenommen:

- (H1) Je höher die Handlungsmotivation ist, TA unter gesundheitsförderlichen Aspekten strategisch im Unternehmen zu verankern, desto stärker die Absicht zur Durchführung operativer Maßnahmen, wie z. B. die Verwendung unterstützender Materialien.

Das TPB-Basismodell postuliert des Weiteren:

- (H2) Die Motivation zur strategischen Verankerung ist umso stärker, (a) je positiver die Einstellung, (b) je unterstützender die sozialen Normen im Unternehmen und (c) je größer die Verhaltenskontrolle von Handlungsträgern gegenüber gesundheitsfördernder TA.

Weil Handlungsabsichten mit Verhaltensüberzeugungen verknüpft sind, leitet das erweiterte Modell folgendes ab:

- (H3) Je bessere telearbeitsbezogene Ergebnisse vorliegen und Erfahrungen gemacht wurden, desto (a) positiver die Einstellung, (b) stärker die sozialen Normen und (c) höher die wahrgenommene Kontrollüberzeugung bzw. Selbstwirksamkeit von Handlungsträgern gegenüber gesundheitsförderlicher TA.

### Fragebogenentwicklung

Ein Online-Workshop mit 11 Expertinnen und Experten aus Wissenschaft und Praxis (BGF/BGM-Beratern von Unternehmen, Netzwerk BGF sowie Forschende zur Arbeitswelt) diente als qualitativer Schritt bei der Entwicklung eines standardisierten Fragebogens. Für das einheitliche Verständnis diente folgende Definition: „Unter gesundheitsförderlicher TA wird verstanden, dass unterschiedliche Rahmenbedingungen berücksichtigt werden, die ein gesundheitsorientiertes Arbeiten zu Hause ermöglichen, z. B. die Einhaltung von Unternehmensrichtlinien, das Führungsverständnis in der virtuellen Zusammenarbeit, ergonomische und ansprechende Gestaltung des Arbeitsplatzes, Verfügbarkeit der notwendigen Arbeitsmittel sowie Hard- und Software, Berücksichtigung personaler Ressourcen und Kompetenzen sowie Aspekten der sozialräumlichen Umgebung.“ In einem Pretest mit 10 Personen wurde das Erhebungsinstrument auf Verständlichkeit, Reihenfolge und Beantwortungszeit der Fragen überprüft.

Die theoretischen Konstrukte wurden mit mehreren Indikatoren, überwiegend mit 5‑stufigen bipolaren Antwortformaten, gemessen:

- Intention wurde gemessen mit „Wie stark/schwach verfolgt das Unternehmen die Strategie, gesundheitsförderliche TA umzusetzen/auszubauen?“ sowie den Fragen „Wie (un)wahrscheinlich ist es …“ bzw. „Wie groß/klein ist die Absicht, dass das Unternehmen den Leitfaden bei der Umsetzung von TA berücksichtigen wird?“ (α = 0,91).
- Zur Einstellungsmessung diente ein Polaritätsprofil: „gesundheitsförderliche TA ist sehr unwichtig/wichtig“, „nutzlos/nützlich“, „schlecht/gut“ (α = 0,93).
- Soziale Normen wurden mit 5 Items gemessen, z. B. „Wie stark oder schwach wird von Ihnen erwartet, dass Sie sich für TA im Unternehmen einsetzen?“ (α = 0,72).
- Verhaltenskontrolle über 3 Variablen, z. B. „Wie groß/klein ist Ihr Einfluss darauf, ob gesundheitsförderliche TA im Unternehmen (weiter)entwickelt wird?“ (α = 0,59).

Hinsichtlich der telearbeitsbezogenen Erfahrungen und Ergebnisse dienten jeweils 2 Items zur TA-Readiness (wie gut das Unternehmen technisch und hinsichtlich der Kommunikation auf TA vorbereitet war; α = 0,82) und zum Anspruch auf TA im Unternehmen (wie vielen Prozent der Belegschaft [0–100 %] während und nach dem ersten Lockdown TA im Unternehmen ermöglicht wurde; α = 0,87) sowie Einzelitems: (a) Ausmaß der vorhandenen TA-Kultur im Unternehmen und (b) Bereitschaft, TA im Unternehmen zu ermöglichen.

Als relevante Hintergrundfaktoren wurden der Wirtschaftssektor (privater vs. öffentlicher/Non-Profit/sonstiger Sektor), die strukturelle und normative Verankerung von BGF/BGM (α = 0,65) und die Betriebsgröße (Großbetrieb > 249 vs. Klein‑/Mittelbetrieben ≤ 249 Beschäftigten) der Unternehmen erfasst.

### Untersuchungsform und Datenerhebung

Zur betrieblichen Unterstützung wurde während des 1. Lockdowns im Frühjahr 2020 ein Link zu einem bestehenden „Leitfaden für gesundheitsförderliche Telearbeitsplätze“ [15] per E‑Mail verschickt und im September um Teilnahme an einer standardisierten Online-Befragung (LimeSurvey GmbH, Hamburg, Deutschland)^3^ gebeten. Zur Auswahlgesamtheit zählten *n* = 1858 Entscheidungsträger:innen, die ein gefördertes BGF-Unternehmensprojekt geleitet/begleitet oder an einer BGF-Fortbildung teilgenommen haben. 192 Betriebe, die TA während des Untersuchungszeitraums umgesetzt hatten, bildete die Analysegrundlage (Rücklauf 10,3 %; Tab. 1).

**Tab. 1:** Charakteristika der Unternehmen und Respondenten

Charakteristika	Kategorien	*n*	%
Unternehmensgröße (*n* = 184)	10–49	44	20,9
	50–249	54	29,3
	> 250 Beschäftigte	86	46,7
Region (Bundesland; *n* = 181)	Ostösterreich (B/NÖ/W)	78	43,0
	Südösterreich (K/ST)	44	24,3
	Westösterreich (OÖ/S/T/V)	59	32,6
Sektor (*n* = 174)	Privater	56	32,2
	Öffentlicher	70	40,2
	Non-Profit	39	22,4
	Sonstiger	9	5,2
BGF-Auszeichnung^a^ (*n* = 192)	Nein/keine Angabe	74	38,5
	BGF-Gütesiegel	103	53,6
	BGF-Preis	15	7,8
Position/Verantwortung (*n* = 303)^b^	BGF/BGM	110	57,3
	Personalabteilung/HRM	48	25,5
	Geschäftsführung	36	19,1
	Präventivdienst^c^	25	13,3
	Arbeitnehmervertretung	18	9,6
	Qualitätsmanagement	15	8,0
	Andere/r	51	27,1

*BGF/BGM* Betriebliche/s Gesundheitsförderung/-management

^a^BGF-Gütesiegel/-Preis des Österreichischen BGF-Qualitätsmanagementsystems

^b^Mehrfachantwortenset: % auf Basis gültiger Fälle

^c^Inklusive Sicherheits‑, Behindertenvertrauensperson, Gleichstellungsbeauftragte

### Statistische Datenanalyse

Die Analyse mittels JASP (University of Amsterdam, Niederlande)^4^ erfolgte in mehreren Schritten: Zur Bildung additiver Skalen wurde eine Reliabilitätsanalyse durchgeführt, wobei zumindest moderate interne Konsistenzreliabilität (Cronbach’s α ≥ 0,60) vorliegen musste [21]. Für Einzelindikatoren und Skalen wurden bedeutsame Streuungen um den Mittelwert, nicht schiefe (s^3^ < |2,0|) sowie unimodale (s^4^ = |7,0|) Verteilungen erwartet ([6]; Tab. 2).

**Tab. 2:** Skalen/Items, Deskriptivstatistiken, interne Konsistenzen und Korrelationen (Pearson r, *n* ≥ 149)

Skalen/Items	MW	SD	s^3^	s^4^	α	1	2	3	4	5	6	7	8	9	10	11
1 Intention Leitfaden	6,00	2,12	−0,16	−0,51	0,91	1,00	–	–	–	–	–	–	–	–	–	–
2 TA-Motivation	2,77	1,09	0,12	−0,64	–	0,57^a^	1,00	–	–	–	–	–	–	–	–	–
3 Einstellung gegenüber TA	13,11	2,06	−1,34	1,50	0,93	0,05	0,13	1,00	–	–	–	–	–	–	–	–
4 Soziale Norm	13,97	2,85	−0,53	0,48	0,72	0,42^a^	0,51^a^	0,25^a^	1,00	–	–	–	–	–	–	–
5 Verhaltenskontrolle	9,21	2,59	−0,14	−0,22	0,59	0,52^a^	0,67^a^	0,08	0,54^a^	1,00	–	–	–	–	–	–
6 TA-Bereitschaft	4,05	0,72	−0,36	−0,12	–	0,07	0,18^a^	0,30^a^	0,40^a^	0,57^a^	1,00	–	–	–	–	–
7 TA-Kultur	2,82	1,09	−0,16	−0,77	–	0,32^a^	0,54^a^	0,00	0,55^a^	0,52^a^	0,19^a^	1,00	–	–	–	–
8 TA-Readiness	6,51	2,23	−0,37	−0,66	0,82	0,35^a^	0,44^a^	−0,08	0,34^a^	0,39^a^	0,17^a^	0,48^a^	1,00	–	–	–
9 TA-Anspruch	49,71	31,44	0,17	−1,25	0,87	0,07	0,33^a^	0,07	0,40^a^	0,32^a^	0,20^a^	0,35^a^	0,17^a^	1,00	–	–
10 Betriebsgröße (Großbetrieb)	1,47	0,50	0,13	−2,00	–	−0,10	0,02	0,03	0,09	−0,02	−0,03	−0,06	−0,06	−0,07	1,00	–
11 Sektor (privater Sektor)	0,32	0,47	0,77	−1,43	–	0,14	0,16^a^	−0,11	0,09	0,11	0,00	0,08	0,33	0,00	−0,11	1,00
12 BGF/BGM-Verankerung	11,96	1,99	−0,40	−0,25	0,65	0,22^a^	0,28^a^	0,05	0,20^a^	0,25^a^	0,08	0,16^a^	0,26	0,00	−0,00	−0,02

*n* gültige Fälle, *MW* Mittelwert, *SD* Standardabweichung, *s*^*3*^ „Skewness“, *s*^*4*^ „Kurtosis“, *α* Cronbach’s Alpha, *TA* Telearbeit, *BGF/BGM* betriebliche/s Gesundheitsförderung/-management

^a^*p* < 0,05

Die Hypothesenprüfung erfolgte mit Pfadmodellen, die mit der robusten Maximum-Likelihood(ML)-Schätzmethode bei annäherungsweise normalverteilten Daten geringeren Umfangs (*n* ≤ 200) unverzerrt geschätzte Modellparameter liefert [7]. Zunächst wurde das Basismodell, dann das erweiterte Modell spezifiziert und zuletzt Hintergrundvariablen zur Effektkontrolle hinzugefügt. Zur Modellprüfung dienten lokale und globale Fitkriterien. Höhe und statistische Signifikanz (*p* ≤ 0,05) der standardisierten Regressionskoeffizienten zur Hypothesenprüfung, absolute und komparative Fitindizes zur Bewertung der Gesamtanpassungsgüte der Modelle: χ^2^-Test (χ^2^/df < 3,0), Root mean square error of approximation (RMSEA) < 0,06, Comparative fit index (CFI) > 0,95 und Standardized room mean squared residual (SRMR) < 0,08 [13, 16]. Residualmatrix und Modifikationsindizes bildeten die Grundlage für plausible Modellverbesserungen.

## Ergebnisse

### Absichten gesundheitsförderlicher Telearbeit

Die befragten Unternehmen verfolgen unterschiedlich stark die Strategie, gesundheitsförderliche TA umzusetzen. Zwei Fünftel (41,0 %) der befragten Unternehmen geben an, dass diese Strategie sehr/eher schwach, bei einem Viertel (25,4 %) hingegen sehr/eher stark verfolgt wird (33,6 % weder noch). Ähnlich verhält es sich bei der Absicht, einen Leitfaden zur gesundheitsförderlichen Gestaltung von TA einzusetzen. Eine positive Wahrscheinlichkeit dafür geben 33,5 % (sehr/eher wahrscheinlich) und die Absicht dazu 28,6 % (eher/sehr groß) an.

### Bestimmungsfaktoren gesundheitsförderlicher Telearbeit

**Abb. 1:**
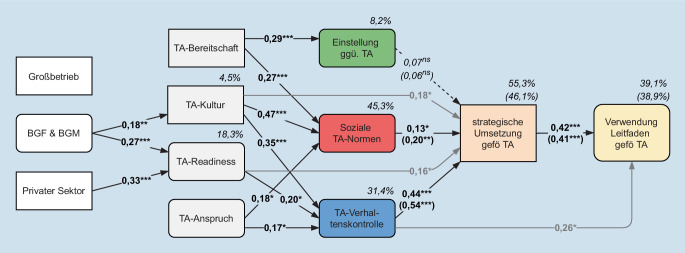
Ergebnisse des Basis- und erweiterten Modells (*n* = 187; *Pfeile* mit standardisierten Effektkoeffizienten [robuste ML-Schätzung], inklusive erklärte Varianz [in %], Ergebnisse des Basismodells in *Klammern*; *p ≤ 0,050, **p ≤ 0,010, ***p ≤ 0,001, *ns* nicht signifikant). Modellfit des Basismodells: χ^2^(df) = 2,50(2) | χ^2^/df = 1,25 | CFI = 0,99 | RMSEA = 0,04 | P‑close = 0,40 | SRMR = 0,02. Fit des erweiterten Modells: χ^2^(df) = 46,55(28) | χ^2^/df = 1,66 | CFI = 0,96 | RMSEA = 0,06 | P‑close = 0,28 | SRMR = 0,05. *TA* Telearbeit, *BGF *betriebliche Gesundheitsförderung,* BGM* betriebliches Gesundheitsmanagement

Mögliche Gründe für diese Absichtserklärungen können in der Einstellung, sozialen Norm und Verhaltenskontrolle gegenüber TA liegen. Beim Basismodell stellt sich die Verhaltenskontrolle bzw. Selbstwirksamkeit als bedeutender Faktor für die Unternehmensstrategie und die sozialen Normen als weitere signifikante förderliche Rahmenbedingung heraus. Hingegen erweisen sich die individuellen Einstellungen statistisch als nicht bedeutend heraus. Die Verwendungsabsicht des TA-Leitfadens wird primär durch die strategische Absicht dazu erklärt und zusätzlich durch die Verhaltenskontrolle. Neben dem direkten ist auch der indirekte Effekt der Verhaltenskontrolle über die strategische Absicht statistisch bedeutend, nicht jedoch der der sozialen Normen (Abb. 1).

Das erweiterte Modell bringt zusätzliche Erkenntnisse: Mit zunehmend stärkerer TA-Kultur im Unternehmen, höherer TA-Bereitschaft und verbreitertem TA-Anspruch während und nach dem 1. Lockdown werden die sozialen Normen gegenüber TA gestärkt. Neben der TA-Kultur und dem TA-Anspruch zeichnet sich die TA-Readiness des Unternehmens (vor dem 1. Lockdown) als einflussreicher Bestimmungsfaktor für die Verhaltenskontrolle aus. Mit stärkerer TA-Bereitschaft im Unternehmen ist außerdem eine positivere Einstellung gegenüber gesundheitsförderlicher TA verbunden. Als zusätzlich bedeutsame direkte Effekte haben sich die TA-Kultur und TA-Readiness für die Absicht der strategischen Umsetzung gesundheitsförderlicher TA herausgestellt.

Nach erfolgter Effektkontrolle muss die Bedeutung der/s implementierten BGF/BGM hervorgehoben werden, die sowohl den Einfluss für die TA-Readiness als auch auf die TA-Kultur belegt. In der Privatwirtschaft tätige Unternehmen waren außerdem besser als andere Unternehmen auf TA vorbereitet, wohingegen keine signifikanten Unterschiede zwischen Betriebsgrößen bestehen.

Alle Modelle werden durch die ausgezeichnete Modellanpassung gestützt und können relevante Anteile der abhängigen Variablen durch die Modellvariablen erklären (Abb. 1).

## Diskussion

Die Ergebnisse verdeutlichen relevante Strukturen und Prozesse hinsichtlich der Implementierung von gesundheitsförderlicher TA im Unternehmen. Die Modelle und Hypothesen (H1, H2b, H2c, H3a–c) konnten überwiegend bestätigt und durch weitere signifikante Effekte ergänzt werden. Die hohen Erklärungsanteile der abhängigen Variablen sind angesichts eines so komplexen Phänomens ein besonders prägnantes Ergebnis.

Am Beispiel von österreichischen Unternehmen konnte gezeigt werden, dass viele Betriebe zum Pandemieausbruch noch zu wenig auf die Umstellung auf TA gerüstet waren. Dementsprechend häufig steht die Absicht für gesundheitsförderliche TA vielerorts noch aus. Neben der strategischen Intention zur Verankerung bedarf es einer operativen Absicht zur Umsetzung von TA (z. B. Verwendung von Praxisleitfäden), wobei diese über die Verhaltenskontrolle von Entscheidungsträgern beeinflusst wird, d. h. die Organisationsentwicklung und Umsetzung in die Praxis setzt eine strategische Entscheidung voraus. Ein wesentlicher Erfolgsfaktor in der BGF ist die Kapazitätsentwicklung in Betrieben. Diese erfordert, geeignete Strukturen aufzubauen, notwendige Ressourcen zu nutzen oder zu mobilisieren sowie adäquate Strategien für die Umsetzung von spezifischen gesundheitsförderlichen Maßnahmen zu entwickeln [12]. Dafür brauchen Entscheidungsträger:innen die nötigen Kapazitäten und Multiplikatorinnen und Multiplikatoren können sowohl auf die strategische Entscheidung als auch die operative Umsetzung Einfluss nehmen. Weitere direkte als auch indirekte Einflussbereiche stellen der Grad der Vorbereitungen auf TA, der Umsetzung von TA während und nach dem Lockdown sowie die TA-Kultur im Unternehmen generell dar.

Betriebliche Normen und kulturelle Aspekte sind Voraussetzungen für gesundheitsförderliche TA, die vom Management gestaltet werden müssen. Die Kultur bestimmt das kollektive organisatorische und das individuelle Verhalten in Organisationen und wirkt sich auf alle Unternehmensbereiche des Managements und ihrer Beschäftigten aus (Entscheidungsfindung, Führung, Sozialbeziehungen, Kommunikation etc.; [18]). Unternehmenskultur stellt sich auch als treibende Kraft für gesundheitsförderliche TA heraus, einerseits direkt über die soziale Norm (sozialer Druck), sich als Verantwortungsträger dafür einzusetzen, und andererseits im Sinne des begünstigenden Sozialklimas (TA-Kultur und TA-Bereitschaft).

Daher erweist sich auch die strukturelle Verfasstheit und normative Verankerung von BGF/BGM im Unternehmen als fundamentaler bzw. begünstigender Hintergrundfaktor. BGF/BGM bestimmt, wie Gesundheit und Wohlbefinden in Grundsätzen und Managementsystemen festgeschrieben (zentralen Unternehmensdokumenten, Leitlinien) und strukturell im Unternehmen verankert ist, entsprechende Verantwortlichkeiten verteilt, Zuständigkeiten und Ansprechpersonen definiert bzw. in Formen der Zusammenarbeit (Empowerment, Partizipation) und (verhältnis- und verhaltensorientierten) Maßnahmen zum Zwecke ihrer Erreichung definiert sind. Nach Schein [22] übt die gesamte (gesundheitsförderliche) Unternehmenskultur als Vorrat kollektiver Überzeugungen, Werte und Regeln nicht nur einen maßgeblichen Einfluss auf die Funktions- bzw. Leistungsbereitschaft einer Organisation aus, sondern auch auf die Leistungsfähigkeit ihrer Mitglieder, d. h. auch auf ihr Wohlbefinden und ihre Gesundheit [3].

Damit bestätigt sich, was bereits Baruch [4] zu organisationalen Bedingungsfaktoren zählte: neben der Art der Arbeit/Tätigkeit und der technischen Eignung bzw. Vorbereitung für spezifische Arbeitsaufgaben ist eine unterstützende Unternehmenskultur und Bereitschaft für wirksame TA unabdingbar. Während die Technologie die Entkoppelung der Arbeit vom Büro erst ermöglicht, sind soziale und organisatorische Faktoren mit der Bereitschaft zur TA verbunden. Neben der nötigen Hard- und Software sind Trainings zur Vermittlung von grundlegendem Wissen und Kenntnissen der IKT und spezifische Fähigkeiten wie Selbstständigkeit, Vertrauen und Kommunikation notwendig.

Die organisatorische Entwicklung zur gesundheitsförderlichen TA erfordert den Aufbau innerbetrieblicher Kapazitäten und Kompetenzen. Dies bewirkt den praktischen Nutzen, Organisationen besser verstehen zu können und ein höheres Bewusstsein über den Einfluss und die Effekte betrieblicher Rahmenbedingungen auf Gesundheit und Wohlbefinden der Beschäftigten zu schaffen.

### Limitationen

Die Ergebnisse sind vor dem Hintergrund methodischer Grenzen einzuordnen:

- Innerbetriebliche Entscheidungsträger:innen stellen nur eine Sicht der Organisationsperspektive dar.
- Retrospektive Fragen können aufgrund des eingeschränkten Erinnerungsvermögens verfälscht sein.
- Eine geringfügig unterschrittene Reliabilität bei der Erhebungsskala Verhaltenskontrolle wurde hier akzeptiert, sollte jedoch in zukünftigen Anwendungen vermieden werden.
- Trotz theoretisch plausibler Wirkketten lassen Querschnittsdesigns streng genommen keine direkten Rückschlüsse kausaler Wirkrichtungen zu.
- Es erfolgte eine bewusste Auswahl von Betrieben und Ergebnisse sollten zukünftig mit einer Zufallsstichprobe repliziert werden.

## Fazit für die Praxis

- Der Beitrag untersucht, wie stark gesundheitsförderliche TA in österreichischen Unternehmen verankert ist und welche Faktoren eine langfristige Etablierung des Ansatzes in Betrieben beeinflussen.
- Die Art der Tätigkeit, BGF-Vorerfahrung, die technischen Möglichkeiten, eine unterstützende Unternehmenskultur, die generelle Bereitschaft für wirksame TA und spezifische individuelle Fähigkeiten beeinflussen die Etablierung von gesundheitsförderlicher TA.
- Die Entwicklung gesundheitsförderlicher TA kann durch entsprechenden Aufbau innerbetrieblicher Kapazitäten und Kompetenzen (z. B. Bewusstsein für eine ganzheitliche Kultur gesundheitsförderlicher TA) und konkrete Maßnahmenüberlegungen für jeden Betrieb begünstigt werden.
- Beispiele dafür können Schulungen für Selbst- und Zeitmanagement, zur virtuellen/hybriden Zusammenarbeit, Führen auf Distanz oder Leitfäden zur Umsetzung von ganzheitlicher gesundheitsförderlicher TA sein.

## Abkürzungen

BGF: Betriebliche Gesundheitsförderung
BGM: Betriebliches Gesundheitsmanagement
CFI: Comparative fit index
COVID-19: Coronavirus disease 2019
df: Degree of freedom
H: Hypothese
IKT: Informations- und Kommunikationstechnologien
ILO: International Labour Organisation
ML: Maximum likelihood
MW: Mittelwert
*n*: Stichprobengröße
*p*: Signifikanzniveau
RMSEA: Root mean square error of approximation
s^3^: Skewness
s^4^: Kurtosis
SD: Standardabweichung
SRMR: Standardized room mean squared residual
TA: Telearbeit
TPB: Theory of planned behavior
WHP: Workplace health promotion
α: Cronbach’s Alpha
χ^2^: χ^2^-Teststatistik

## Footnotes

1 Der Beitrag beschäftigt sich mit Telearbeit im Sinne der Arbeit im privaten Haushalt (Arbeiten im Homeoffice). Neben einer begrifflichen Vielfalt ist darauf hinzuweisen, dass das Phänomen in verschiedenen Ländern gesetzlich unterschiedlich geregelt ist und mitunter Konsequenzen für die Verantwortung des Arbeits- und Gesundheitsschutzes mit sich bringen kann. Im deutschen Arbeitsrecht wird bspw. zwischen Telearbeit und mobiler Arbeit im Homeoffice unterschieden.

2 vgl. European Network for Workplace Health Promotion (www.enwhp.org, abgerufen: 04.12.2021).

3 https://community.limesurvey.org/

4 vgl. https://jasp-stats.org/ (abgerufen: 04.12.2021).
